# Indoor Autonomous Control of a Two-Wheeled Inverted Pendulum Vehicle Using Ultra Wide Band Technology

**DOI:** 10.3390/s17061401

**Published:** 2017-06-15

**Authors:** Dunzhu Xia, Yanhong Yao, Limei Cheng

**Affiliations:** Key Laboratory of Micro-inertial Instrument and Advanced Navigation Technology, Ministry of Education, School of Instrument Science and Engineering, Southeast University, Nanjing 210096, China; 220142708@seu.edu.cn (Y.Y.); 220152675@seu.edu.cn (L.C.)

**Keywords:** two-wheeled vehicle, indoor, UWB, nonlinear, second-order sliding mode control (2-SMC), robust adaptive

## Abstract

In this paper, we aimed to achieve the indoor tracking control of a two-wheeled inverted pendulum (TWIP) vehicle. The attitude data are acquired from a low cost micro inertial measurement unit (IMU), and the ultra-wideband (UWB) technology is utilized to obtain an accurate estimation of the TWIP’s position. We propose a dual-loop control method to realize the simultaneous balance and trajectory tracking control for the TWIP vehicle. A robust adaptive second-order sliding mode control (2-RASMC) method based on an improved super-twisting (STW) algorithm is investigated to obtain the control laws, followed by several simulations to verify its robustness. The outer loop controller is designed using the idea of backstepping. Moreover, three typical trajectories, including a circle, a trifolium and a hexagon, have been designed to prove the adaptability of the control combinations. Six different combinations of inner and outer loop control algorithms have been compared, and the characteristics of inner and outer loop algorithm combinations have been analyzed. Simulation results demonstrate its tracking performance and thus verify the validity of the proposed control methods. Trajectory tracking experiments in a real indoor environment have been performed using our experimental vehicle to further validate the feasibility of the proposed algorithm in practice.

## 1. Introduction

Two-wheeled inverted pendulum (TWIP) vehicles are a popular test platform to validate the performance of advanced control algorithms, such as the control capacity for nonlinear, instability and rapidity situations. The motion of a TWIP vehicle is governed by its under-actuated configuration, which makes it difficult to control by simply applying traditional robotics approaches [1]. In recent years, control design for the stability and robustness of TWIP vehicles has attracted great research interest. In [2], a neural network control method was proposed for an under-actuated wheeled inverted pendulum model with small overshoot and quick response to changes in the desired trajectories. In [3], adaptive backstepping control for a two-wheeled autonomous robot around an operating point was investigated. In [4], an adaptation concept was brought into neural network control, which demonstrated the yaw angle and pitch angle convergence time was less than 0.4 s with an initial value of 0.3 rad.

The sliding mode control (SMC) method was first proposed by Soviet scholars in the 1960s. The SMC method has some advantages such as robustness to parameter uncertainty, insensitivity to bounded disturbances, fast dynamic response, a remarkable computational simplicity with respect to other robust control approaches, and easy implementation of the controller [5,6]. However, the chattering caused by the discontinuous sliding controller more or less limits its application in practice [7].

The high-order sliding mode control (HOSMC) method is the further improvement of the traditional sliding mode control, yielding less chattering and better convergence accuracy while preserving its robustness properties [8,9]. Consequently, HOSMC methods have been actively investigated in recent years for chattering attenuation and robust control of unknown uncertainties and perturbations, respectively. Levant put forward the 2-SMC method based on a differentiation and output-feedback control system in [10], and there are many different 2-SMC algorithms that have been presented such as the ‘twisting’ and ‘super-twisting’ ones put forward by Levant, the ‘sub-optimal’ and ‘global’ ones developed by Bartolini and so on.

The objective of this paper is to sustain the self-balance and trajectory tracking of a TWIP vehicle in a GPS denied environment. Nakano et al. have proposed an estimation/control structure in a two-wheeled vehicle where both a camera and a target object are attached to the vehicle [11]. The major drawback of the approach based on camera is that it may produce a heavy computational load and lead to a low real-time performance. Accuracy and precision are the two main performance parameters of the indoor localization system. Portable UWB radio ranging technology was introduced in [12,13]. The UWB local positioning system can be used as a standalone system or as a complementary system where the GPS is unavailable or denied, can meet high real-time requirements and even penetrate through objects (such as walls, obstacles, bodies, etc.) to a certain extent.

**Figure 1:**
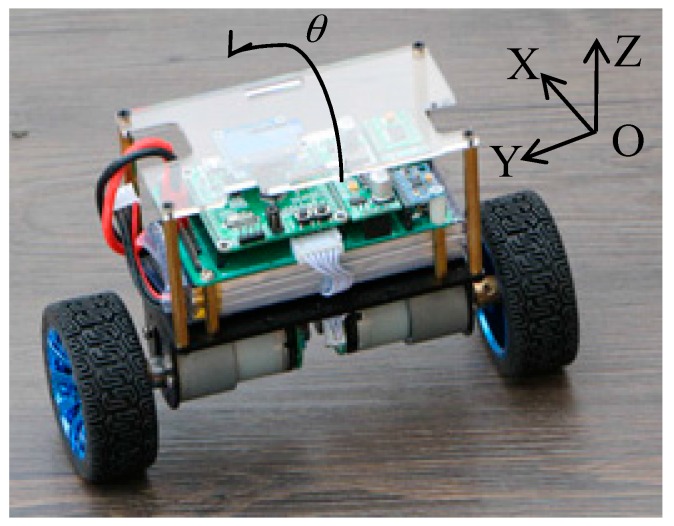
The experimental two-wheeled inverted pendulum vehicle.

In this paper, a prototype TWIP vehicle (shown in Figure 1) was built in our lab. The TWIP vehicle consists of two independent drive wheels on the same axle, two rotary encoders, a car body and other auxiliary devices such as a sensor (MPU-6050) which has an embedded 3-axis MEMS gyroscope, a 3-axis MEMS accelerometer (MEMS sensors have the advantages of miniaturization and high-performance [14]) and a portable I-UWB locating tag.

**Figure 2:**
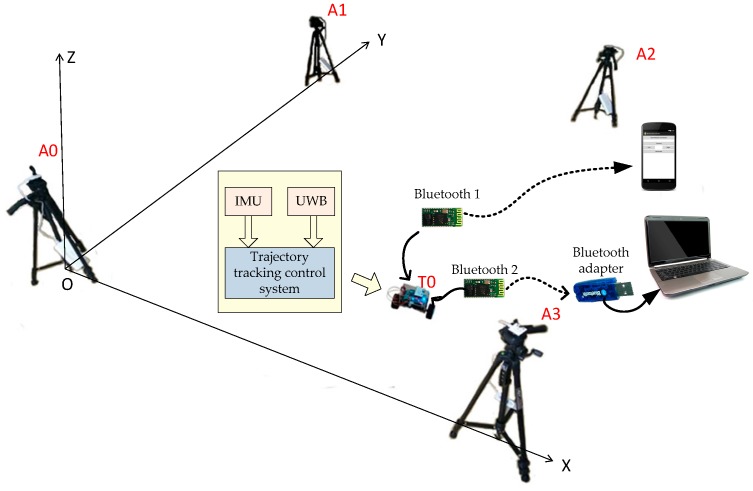
System architecture.

The control system configuration of our testbed is illustrated in Figure 2. The attitude data of the TWIP vehicle is measured via the onboard IMU, the location information of the TWIP vehicle is measured by an onboard UWB receiver and used to control the motion of the vehicle. In Figure 2, A0, A1, A2 and A3 are four UWB positioning technology anchors, T0 is a UWB receiver used as an onboard tag. The TWIP vehicle can communicate with both cellphones and laptops via Bluetooth devices.

This paper is organized as follows: in Section 2, the nonlinear dynamic model of the TWIP vehicle is established using a Lagrange energy equation. In Section 3, inner and outer loop controllers are designed, and necessary stability considerations are discussed. Then the feasibility and effectiveness of the different control methods are compared by MATLAB simulation in Section 4, followed by the conclusions in Section 5. The configuration of an experimental set-up of the TWIP vehicle and the results are given in Section 6.

## 2. Related Work

A second-order adaptive sliding mode control method based on STW algorithm was proposed for the control of an under-actuated system in [15], which performed better effectiveness and reduced chattering compared with conventional SMC and ordinary second-order SMC. However, the adaptive sliding mode control method is not very effective for a controller of multi-control parameters. In [16], the structure of the inner/outer loop control system has been used in simultaneous balancing and trajectory tracking control for two-wheeled inverted pendulum vehicles, and two trajectories, including a circle and a trifolium have been designed for tracking. For the purpose of controlling the TWIP vehicle, an inner/outer loop control structure and a second-order STW algorithm have been also used in this paper. We proposed a different adaptive control method in this paper to keep convergence of sliding mode surface under unknown disturbances. What’s more, three typical trajectories, including a circle, a trifolium and a hexagon, have been designed to prove the adaptability of our controller.

## 3. System Description and Modeling

**Figure 3:**
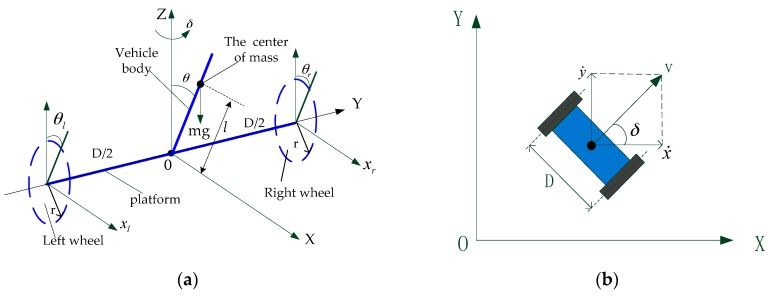
The fixed coordinate system of the vehicle: (**a**) A simplified schematic diagram; (**b**) Motion of the vehicle on the x-y plane.

The motion of the TWIP vehicle is described under an inertial coordinate system. The simplified schematic diagram and the two-dimensional plan view of the vehicle are shown in Figure 3.

The origin O of the fixed coordinate system OXY is the center of the axle, the positive direction of OX-axis is the forward motion of TWIP, OY-axis is coincident with the axle, the positive direction of OZ-axis is vertically upward. The parameters and variables of the system are shown in Table 1. During the motion process of our two-wheeled vehicle, since there are only two points contacting with the ground, and the body has a motion similar to inverted pendulum motion, we can make a few assumptions in order to build dynamic model for the system:

(1) There exists sufficient friction between the wheels and the ground, which makes the system subject to non-holonomic constraints, i.e., the assumption of no sliding and skidding holds throughout.
(2) Take the TWIP vehicle as a perfectly rigid body, the distance from the center of mass of the body to the axle is l;
(3) The influence caused by the dynamic characteristics of DC motors is ignored;
(4) Ignore the effects caused by friction and damping force of bearings and revolute pairs to the motion state of the system.

In the paper, we adopt the Lagrange energy equation to build the kinematic model of the two-wheeled car. In order to eliminate the effect caused by the non-holonomic constraint, we choose $q\;=\;{\left[\theta_{l}\;\theta_{r}\;\theta \right]}^{T}$ as the generalized coordinates system, then the kinematic model of the two-wheeled car can be written in the following form:

(1) $$M\left(q\right)\ddot{q}+V\left(q,\dot{q}\right)\dot{q}+G\left(q\right)=B\left(q\right)u$$

where, $M\left(q\right)\;\in {\;R}^{3\times 3}$ denotes the inertia matrix, $V\left(q,\dot{q}\right)\;\in {\;R}^{3\times 3}$ is the vector of coriolis and centrifugal forces $G\left(q\right)\;\in {\;R}^{3}$ is the vector of gravitational forces, $B\left(q\right)\;\in {\;R}^{3\times 2}$ denotes the matrix of control coefficients, $u\;\in {\;R}^{2}\;$is the vector of control inputs.

Defining a new velocity vector $\dot{\xi }={\left[\dot{\delta }\;{\dot{x}}_{v}\right]}^{T}={\left[\omega \;v\right]}^{T}$, then the relationship between a new coordinate vector $\dot{z}={\left[\dot{\xi }\;\dot{\theta }\right]}^{T}={\left[\omega \;v\;\dot{\theta }\right]}^{T}$ and the generalized coordinate vector $q\;=\;{\left[\theta_{l}\;\theta_{r}\;\theta \right]}^{T}$ can be written as follows:

(2) $$\dot{q}=H\dot{z}=\left[\begin{array}{lll} -\frac{D}{2r} & \frac{1}{r} & 0\\ \frac{D}{2r} & \frac{1}{r} & 0\\ 0 & 0 & 1 \end{array}\right]\dot{z}$$

Then the dynamics of the system can be formulated as follows:

(3) $$\bar{M}\left(z\right)\dot{z}+\bar{V}\left(z,\dot{z}\right)z+\bar{G}\left(z\right)=\bar{B}\left(z\right)u$$

where:

$$\bar{M}\left(z\right)=\left[\begin{array}{ccc} \alpha +ml^{2}\sin^{2}\theta & 0 & 0\\ 0 & Qr & mlr\cos\theta \\ 0 & mlr\cos\theta & P \end{array}\right],\bar{V}\left(z,\dot{z}\right)=\left[\begin{array}{ccc} -ml^{2}\sin2\theta \dot{\theta } & 0 & 0\\ 0 & 0 & -mlr\sin\theta \dot{\theta }\\ -\frac{1}{2}ml^{2}\sin2\theta \dot{\delta } & 0 & 0 \end{array}\right],$$

$$\bar{G}\left(z\right)=\left[\begin{array}{c} 0\\ 0\\ -mgl\sin\theta \end{array}\right],\;\bar{B}\left(z\right)=\left[\begin{array}{cc} -\frac{D}{r} & \frac{D}{r}\\ \frac{r}{2} & \frac{r}{2}\\ -1 & -1 \end{array}\right],u=\left[\begin{array}{c} \tau_{l}\\ \tau_{r} \end{array}\right],$$

$$P=ml^{2}+J_{\theta },\;Q=2M_{w}+\frac{2J_{w}}{r^{2}}+m,$$

$$\alpha =D^{2}\left(2M_{w}+\frac{J_{w}}{r^{2}}\right)+J_{\delta },\;\beta =\mathrm{PQ}-m^{2}l^{2}\cos^{2}\theta$$

In order to simplify the controller design, the state-space equations can be derived from (3):

(4) $$\dot{\omega }=f_{1}\left(x\right)+g_{1}\left(x\right)\tau_{\omega }$$

(5) $$\dot{v}=f_{2}\left(x\right)+g_{2}\left(x\right)\tau_{v}$$

(6) $$\ddot{\theta }=f_{3}\left(x\right)+g_{3}\left(x\right)\tau_{v}$$

where:

$$f_{1}\left(x\right)=-\frac{ml^{2}\sin2\theta \dot{\theta }\dot{\delta }}{\alpha +ml^{2}\sin^{2}\theta },\;g_{1}\left(x\right)=\frac{D}{r\left(\alpha +ml^{2}\sin^{2}\theta \right)},$$

$$f_{2}\left(x\right)=\frac{1}{2\beta }(-m^{2}gl^{2}\sin2\theta -m^{2}l^{3}\sin2\theta \cos\theta {\dot{\delta }}^{2}+2Pml\sin\theta {\dot{\theta }}^{2}),\;g_{2}\left(x\right)=\frac{P+mlr\cos\theta }{\beta r},$$

$$f_{3}\left(x\right)=\frac{1}{2\beta }(-m^{2}l^{2}\sin2\theta {\dot{\theta }}^{2}+Qml^{2}\sin2\theta {\dot{\delta }}^{2}+2Qmgl\sin\theta ),\;g_{3}\left(x\right)=-\frac{Qr+ml\cos\theta }{\beta r},$$

$x={\left(\delta \;\omega \;x_{v}\;v\;\theta \;\dot{\theta }\right)}^{T}$ is known as the system state. $u={\left(\tau_{\omega }\;\tau_{v}\right)}^{T}$ is the system control input, and $\tau_{\omega }=\tau_{r}-\tau_{l}$, $\tau_{v}=\tau_{r}+\tau_{l}$.

## 4. Design of the Controller

**Figure 4:**
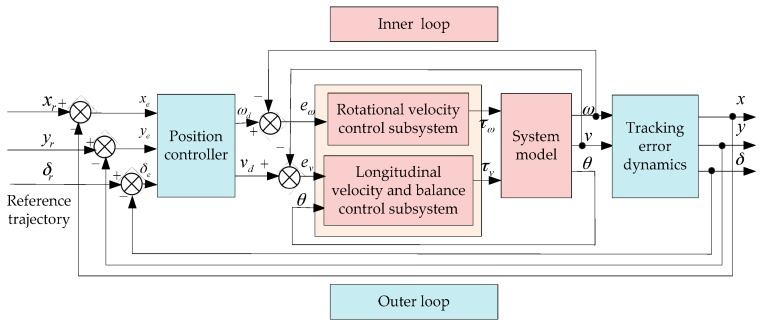
The structure of the inner/outer loop control system.

An inner/outer loop controller needs to be designed to make the vehicle achieve trajectory tracking on the ground while maintaining its own balance. The structure of the designed inner/outer loop control system is shown in Figure 4. The inner loop control consists of two control subsystems: the rotational velocity control subsystem and the longitudinal velocity and balance control subsystem. $v_{d}$, $\omega_{v}$ are defined as the desired value of the longitudinal velocity v and the rotational velocity ω respectively. And the tracking errors of v and ω are introduced as $e_{v}=v-v_{d}$, $e_{\omega }=\omega -\omega_{d}$. A reference trajectory is known in prior as $x_{r}(t)$, $y_{r}(t)$, $\delta_{r}(t)$, and the control laws of $v_{d}$ and $\omega_{d}$ are given by the outer loop controller.

### 4.1. Inner Loop Controller Design

Considering the desired inclination angle $\theta_{d}$ = 0, a set of error state-space equations can be obtained as follows:

(7) $${\dot{e}}_{\omega }=f_{1}\left(x\right)+g_{1}\left(x\right)\tau_{\omega }-{\dot{\omega }}_{d}$$

(8) $${\dot{e}}_{v}=f_{2}\left(x\right)+g_{2}\left(x\right)\tau_{v}-{\dot{v}}_{d}$$

(9) $$\ddot{\theta }=f_{3}\left(x\right)+g_{3}\left(x\right)\tau_{v}$$

#### 4.1.1. The Rotational Velocity Control

It is well known that the classical discontinuous sliding mode control provides finite-time convergence for a system of relative degree one. As the rotational velocity control subsystem (7) has a relative degree equal to two, a sliding surface is needed when using STW algorithm.

The tracking error of δ is introduced as $e_{\delta }=\delta_{r}-\delta$. And the equilibrium point of system (7) can be defined as $X_{e1}={\left[e_{\delta }\;e_{\omega }\right]}^{T}={\left[0\;0\right]}^{T}$. Defining a traditional linear sliding surface $s_{1}$ as:

(10) $$s_{1}=c_{1}e_{\delta }+e_{\omega }$$

where, $c_{1}$ is a positive constant. Differentiating (10) with respect to time, it follows that:

(11) $${\dot{s}}_{1}=c_{1}{\dot{e}}_{\delta }+{\dot{e}}_{\omega }=c_{1}e_{\omega }+f_{1}\left(x\right)+g_{1}\left(x\right)\tau_{\omega }-{\dot{\omega }}_{d}=\varphi_{1}\left(x\right)+g_{1}\left(x\right)\tau_{\omega }$$

where, $\varphi_{1}\left(x\right)=c_{1}e_{\omega }+f_{1}\left(x\right)-{\dot{\omega }}_{d}$.

The continuous control law $\tau_{w}$ is constituted by two terms. The first is defined by means of its discontinuous time derivative, while the other is a continuous function of the available sliding surface $s_{1}$.

Assume that there exists a bounded time-variable disturbance signal $d_{1}\left(t\right)$ in the system (7), which satisfies $\left|{\dot{d}}_{1}\left(t\right)\right|\leq L$, *L* is a Lipschitz constant. According to the STW algorithm [10], the control law of $\tau_{\omega }$ can be obtained as follows:

(12) $$\begin{array}{l} \tau_{\omega }=(-\varphi_{1}\left(x\right)-k_{1}{\left|s_{1}\right|}^{\frac{1}{2}}sign\left(s_{1}\right)+v_{1})/g_{1}\left(x\right)\\ {\dot{v}}_{1}=-k_{2}sign\left(s_{1}\right)+{\dot{d}}_{1} \end{array}$$

**Theorem** **1.** According to the convergent method proposed in [17], when $k_{1}>2$, $k_{2}>\frac{k_{1}^{3}+(4k_{1}-8)L^{2}}{k_{1}(4k_{1}-8)}$, the states of system (7) can reach $s_{1}={\dot{s}}_{1}=0$ in finite time, and converge to origin.

#### 4.1.2. The Longitudinal Velocity and Balance Control

We define $x_{vd}$, $v_{d}$ as the expected values of longitudinal displacement $x_{v}$ and longitudinal velocity *v* respectively and consider the tracking errors of $x_{vd}$, $v_{d}$ as $e_{xv}=x_{v}-x_{vd}$, $e_{v}=v-v_{d}$. The equilibrium point of systems (8) and (9) can be defined as $X_{e2}={\left[e_{xv}\;e_{v}\;\theta \;\dot{\theta }\right]}^{T}={\left[0\;0\;0\;0\right]}^{T}$. The control law of $\tau_{v}$ is designed to make the vehicle accomplish the ultimate objective, that is, when *t → ∞*, limθ=0 as well as $limv=v_{d}$.

Due to its ingenious design, simple algorithm structure and good control effect, hierarchical sliding mode control theory is widely used in complex under-actuated systems control [18,19,20]. Therefore, we adopt the hierarchical sliding mode surface to design the control law of $\tau_{v}$.

In order to design control strategy, two traditional linear sliding mode surfaces with known positive constants c_2_ and c_3_ are defined as:

(13) $$\left\{ \begin{array}{l} s_{2}=c_{2}e_{xv}+e_{v}\\ s_{3}=c_{3}\theta +\dot{\theta } \end{array}\right.$$

Differentiating s_2_ and s_3_ with respect to time, we can obtain:

(14) $$\left\{ \begin{array}{l} {\dot{s}}_{2}=c_{2}e_{v}+{\dot{e}}_{v}\\ {\dot{s}}_{3}=c_{3}\dot{\theta }+\ddot{\theta } \end{array}\right.$$

A control law based on piecewise linear hierarchical sliding mode control method needs to be changed occasionally, bringing in severe oscillation during the control process. In view of the decoupled sliding-mode control method introduced in [21], a hierarchical sliding mode control method based on model analysis is proposed.

The first layer sliding mode surface s_2_ and s_3_ is used to construct the second layer sliding mode surface S. Define the second layer sliding mode surface S as:

(15) $$S=\alpha s_{2}+s_{3}\;\mathrm{with}\;\alpha \;>\;0$$

Differentiating (15) with respect to time and then:

(16) $$\begin{array}{l} \dot{S}=\alpha c_{2}e_{v}+c_{3}\dot{\theta }+\alpha f_{2}\left(x\right)+f_{3}\left(x\right)-{\dot{v}}_{d}+\left(\alpha g_{2}\left(x\right)+g_{3}\left(x\right)\right)\tau_{v}\\ \;=\varphi_{2}\left(x\right)+\psi (x)\tau_{v} \end{array}$$

where $\varphi_{2}\left(x\right)=\alpha c_{2}e_{v}+c_{3}\dot{\theta }+\alpha f_{2}\left(x\right)+f_{3}\left(x\right)-{\dot{v}}_{d}$ and $\psi \left(x\right)=\alpha g_{2}\left(x\right)+g_{3}\left(x\right)$ are Lipschitz continuous.

Assume that there exists a bounded time-variable disturbance signal $d_{2}\left(t\right)$ in the systems (8) and (9), which satisfies $\left|{\dot{d}}_{2}\left(t\right)\right|\leq L_{2}$, $L_{2}$ is a Lipschitz constant. Similar to the analysis of rotational angle control, the 2-SMC control law for the longitudinal velocity and balance control subsystems (8) and (9) can be deduced as follows:

(17) $$\begin{array}{l} \tau_{v}=(-\varphi_{2}\left(x\right)-k_{3}{\left|S\right|}^{\frac{1}{2}}sign\left(S\right)+v_{2})/\psi \left(x\right)\\ {\dot{v}}_{2}=-k_{4}sign\left(S\right)+{\dot{d}}_{2} \end{array}$$

where, S is the sliding mode surface of the control subsystem.

**Theorem** **2.** According to Theorem 1, when $k_{3}\;>\;2,\;k_{4}\;>\frac{k_{3}^{3}+\left(4k_{3}-8\right){L_{2}}^{2}}{k_{3}\left(4k_{3}-8\right)}$, the states of subsystems (8) and (9) can reach $S=\dot{S}=0$ and converge to origin in finite time with the control law (17).

**Proof.**  The process to prove the subsystems (8) and (9) can reach $S=\dot{S}=0$ is similar to Theorem 1’s, so for simplicity, the proof is omitted here. Suppose that $s_{2}\;>0$ at a moment before the system return back to the equilibrium position. A negative control input $\tau_{v}$ is given to push $s_{2}$ to zero, that is,$\dot{{\;s}_{2\;}}<0$. At this moment, under the same negative control input, $s_{3}\;<0$ and $\dot{{\;s}_{3}}\;>0$. Since α is chosen as a positive constant, when S converges to 0, $s_{2}$ and $s_{3}$ can always satisfy the condition that $s_{2}\;>0$, $s_{3}\;<0$, and gradually converge to 0 and vice versa. It can be concluded that subsystems (8) and (9) will converge to $e_{xv}=e_{v}=\theta =\dot{\theta }=0$ in exponential form. ☐

#### 4.1.3. Robust Adaptive 2-SMC (2-RASMC)

Considering the rotational velocity control subsystem (7), when $f_{1}\left(x\right){,\;g}_{1}\left(x\right)$ are unknown functions, defining a new coordinate vector ${z=s}_{1}$, the system model can be written as the following form:

(18) $$\begin{array}{l} \dot{z}=f_{1}\left(x\right)-{\dot{\omega }}_{d}+c_{1}e_{\omega }+g_{1}\left(x\right)\tau_{\omega }=\varphi \left(x\right)+g_{1}\left(x\right)\tau_{\omega }\\ \;=\varphi \left(x\right)+(g_{1}\left(x\right)-1)\tau_{\omega }+\tau_{\omega }=\psi \left(x\right)+\tau_{\omega } \end{array}$$

where $g_{1}\left(x\right)$ is assumed strict positivity without loss of generality, ${\varphi (x)\;=\;f}_{1}\left(x\right)\;-{\dot{\omega }}_{d}{\;+\;c}_{1}e_{\omega }$ and $g_{1}\left(x\right)$ are bounded unknown functions, and satisfying the following inequalities with positive constants $C\;\geq \;0,\;\Gamma_{m}\;>\;0,\;\Gamma_{M}\;>\;0$:

(19) |φ(x)|≤C

(20) $$0<\Gamma_{m}<g_{1}\left(x\right)<\Gamma_{M}$$

As the system model contains uncertain part, the control input $\tau_{w}$ can be designed as two parts:

(21) $$\tau_{\omega }=\tau_{\omega nom}+\tau_{\omega com}$$

where, $\tau_{\mathrm{wnom}}$ is the nominal part of $\tau_{w}$, and $\tau_{\mathrm{wcom}}$ is the compensation control part of $\tau_{w}$.

The nominal system model (ψ(x) = 0) can be obtained as:

(22) $$\dot{z}=\tau_{wnom}$$

According to Theorem 2, the control system (22) is asymptotically stable and reach $z=\dot{z}=0$ within finite time with the following control law of $\tau_{\mathrm{wnom}}\;\left(23\right)$:

(23) $$\left\{ \begin{array}{l} \tau_{wnom}=-k_{1}{\left|z\right|}^{\frac{1}{2}}sign\left(z\right)+v_{3}\\ {\dot{v}}_{3}=-k_{2}sign\left(z\right) \end{array}\right.k_{1}>0,k_{2}>0,$$

In view of an adaptive sliding mode control method introduced in [19], an adaptive sliding mode control law of $\tau_{\mathrm{wcom}}$ is proposed, with which the system perturbation can be compensated. Firstly, an auxiliary variable $z_{\mathrm{aux}}\;\in \;R$ is introduced and satisfying ${\dot{z}}_{\mathrm{aux}}\;=\;-\tau_{\omega \mathrm{com}}$, then define a sliding mode variable σ(z) ∈ R as:

(24) $$\sigma \left(z\right)=z+z_{aux}$$

Differentiating (24) with respect to time, it is easily obtained that:

(25) $$\begin{array}{ll} \dot{\sigma }\left(z\right) & =\dot{z}+{\dot{z}}_{aux}=\varphi \left(x\right)+g_{1}\left(x\right)\tau_{\omega }-\tau_{\omega nom}\\ & =\varphi \left(x\right)+\left(g_{1}\left(x\right)-1\right)\tau_{\omega }+\tau_{\omega nom}=a(x,\tau_{\omega })+\tau_{\omega com} \end{array}$$

where, ${a(x,\;\tau }_{\omega })\;{=\;\varphi (x)\;+\;(g}_{1}(x)\;-\;{1)\tau }_{\omega }$ is the total uncertainty of the system, and satisfying $\left|a\left({x,\;\tau }_{\omega }\right)\right|\;<r_{1}$, where $r_{1}$ is an unknown positive constant. Let us consider ${\hat{r}}_{1}$ as the estimation of true value $r_{1}$, and consequently an estimated error can be governed by ${\tilde{r}}_{1}\;=\;{\hat{r}}_{1}-r_{1}$. In addition, considering the fact that ${a(x,\;\tau }_{\omega })$ is exhibiting slow time-varying behaviors, then, it holds that ${\dot{\tilde{r}}}_{1}\approx \;{\hat{r}}_{1}$.

The compensation control law for the subsystem can be given by:

(26) $$\tau_{\omega com}=-{\hat{r}}_{1}\mathrm{sgn}(\sigma )$$

with the adaptive law as follows:

(27) $${\dot{\hat{r}}}_{1}=\frac{1}{\gamma_{1}}\left|\sigma \right|,\gamma_{1}>0$$

**Theorem** **3.** Consider the given dynamic sliding mode (24) with the nominal controller (23) and the compensation controller(26), the sliding mode variable σ = 0 can be guaranteed in finite time.

**Proof.**  Consider a positive Lyapunov candidate as:

(28) $$V=\frac{1}{2}\sigma^{2}+\frac{1}{2}\gamma_{1}{{\tilde{r}}_{1}}^{2}$$

By differentiating V with respect to time and considering ${\dot{\tilde{r}}}_{1}\;\approx \;{\dot{\hat{r}}}_{1}$, it holds that:

(29) $$\begin{array}{ll} \dot{V} & =\sigma \dot{\sigma }+\gamma_{1}({\hat{r}}_{1}-r_{1}){\dot{\hat{r}}}_{1}=\sigma \left(a(x,\tau_{w})-{\hat{r}}_{1}\mathrm{sgn}\left(\sigma \right)\right)+({\hat{r}}_{1}-r_{1})\left|\sigma \right|\\ & \leq -r_{1}\left|\sigma \right|+\left|\sigma \right|\left|a(x,\tau_{w})\right|\leq -\left|\sigma \right|\left(r_{1}-\left|a(x,\tau_{w})\right|\right) \end{array}$$

It can be seen that $\dot{V}\leq 0$ for ∀t∈(0,+∞). By LaSalle's invariance principle [22,23] and the Lyapunov stability criterion [24], the theorem is proved.

Similar to the above analysis, we can deduce the robust adaptive 2-SMC control law for the longitudinal velocity and balance control subsystems (8) and (9) as follows:

(30) $$\tau_{v}=\tau_{vnom}+\tau_{vcom}$$

(31) $$\left\{ \begin{array}{l} \tau_{vnom}=-k_{3}{\left|S\right|}^{\frac{1}{2}}sign\left(S\right)+v_{4}\\ {\dot{v}}_{4}=-k_{4}sign\left(S\right)\\ \sigma_{2}=S+z_{aux2}\;\mathrm{with}\;{\dot{z}}_{aux2}=-\tau_{vnom}\\ \tau_{vcom}=-{\hat{r}}_{2}\mathrm{sgn}(\sigma_{2})\;\mathrm{with}\;{\dot{\hat{r}}}_{2}=\frac{1}{\gamma_{2}}\left|\sigma_{2}\right| \end{array}\right.,\;k_{3}>0,k_{4}>0,\gamma_{2}>0$$

where, S is the sliding mode surface (15) of the control subsystem. ☐

**Theorem** **4.** According to Theorems 2 and 3, when $k_{3}>0,\;k_{4}>0,\;\gamma_{2}>0$, the state of systems (8) and (9) can reach $S\;=\dot{\;S}\;=\;0\;$ with the control law (31), and converge to origin in finite time. The proof process is similar with above and is omitted here.

#### 4.1.4. Comparative Simulations for the Robust Performance

In this section, a comparative simulation has been done to verify the robustness of the modified 2-RASMC control method. Since the 2-RASMC controller is based on the 2-SMC controller, they share the same control parameters, except two additional parameters $\gamma_{1}$ and $\gamma_{2}$. We have done a lot of simulations to find the appropriate control parameters of the designed controller.

Set $\omega_{d}\;=\;0.5\;$rad/s, $v_{d}\;=\;0.5\;$m/s as the control target for the 2-RASMC controller. Then the initial state variables are supposed as ${\left[\delta (0)\;\omega (0)\;x_{v}(0)\;v(0)\;\theta (0)\;\dot{\theta }(0)\right]}^{T}=$ ${\left[0\;0\;0\;0\;0.1\;0\right]}^{T}$.

**Figure 5:**
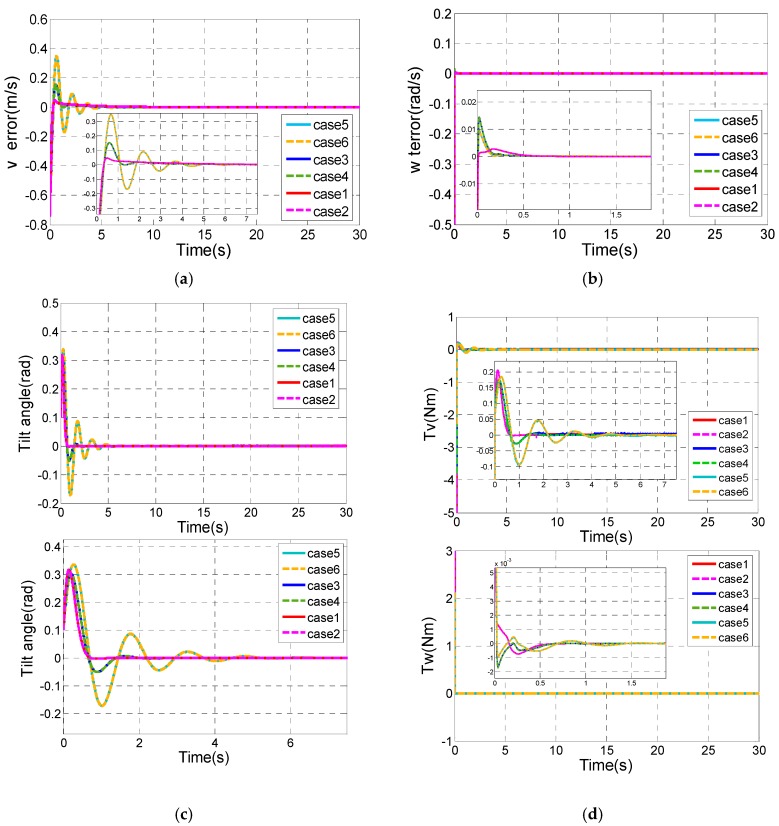
Comparison results of the controller with different control parameters. (**a**) v tracking error; (**b**) ω tracking error; (**c**) Tilt angle of the TWIP vehicle; (**d**) The longitudinal control input $\tau_{v}$ and the rotational control input $\tau_{\omega }$.

Figure 5 depicts the control performance of our controller for tracking the descried $\omega_{d}$ and $v_{d}$ with six different sets of designed parameters as Table 2 shows. Figure 5 shows the control performance of case1~case6 without system uncertainty. It can be concluded that the controllers have the same control performance when the control parameters of the nominal controllers are the same. It can be easily obtained that case 1 and case 2 have the best control performance.

During the process of tuning the control parameters, we have found the effects of the controller parameters on control performance. The increases of $c_{1}$, α, $k_{1}$, $k_{2}$, $k_{3}$ and $k_{4}$ contribute to the faster convergence speed, and make overshoot volume increase. However, the increase of $c_{2}$ contributes to bigger overshoot volume and longer settling time of θ, and bigger overshoot volume and shorter settling time of v. The increase of $c_{3}$ makes overshoot volume of θ decrease, overshoot volume of v increase, and slows down the convergence speed of θ and v.

Then we will consider the control performance of case1 and case2 with system uncertainty. We assumed that the mass of the body is considered as a variable value in the simulation:

$$m=\left\{ \begin{array}{l} 2,t\leq 10\;s\\ 5,t>10\;s \end{array}\right.$$

**Figure 6:**
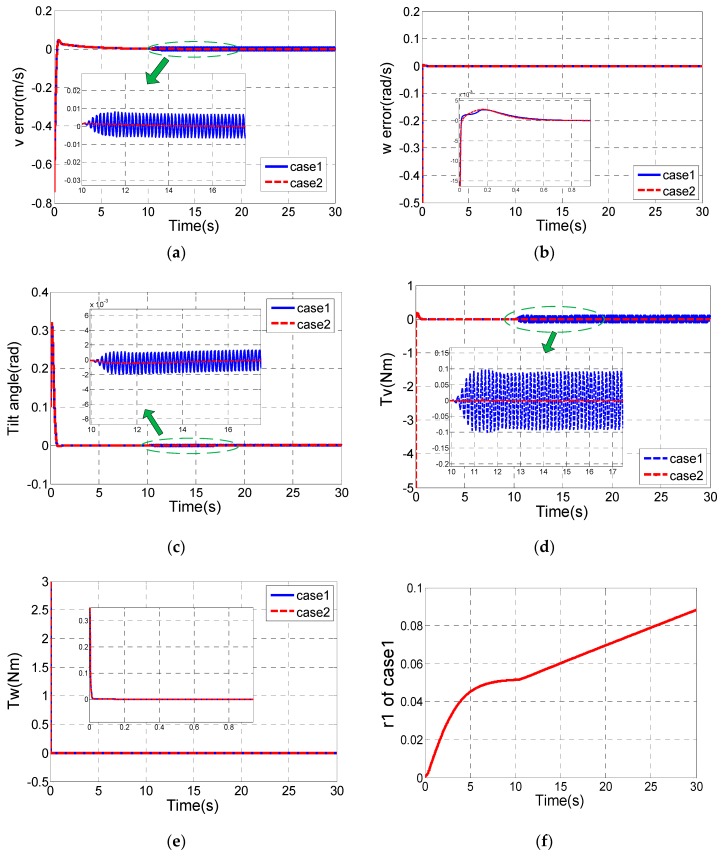

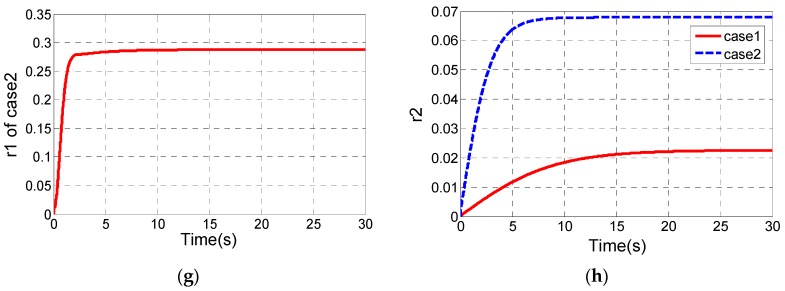
The control performances of case1 and case2 with system uncertainty. (**a**) v tracking error; (**b**) ω tracking error; (**c**) Tilt angle of the TWIP vehicle; (**d**) The longitudinal control input $\tau_{v}$; (**e**) The rotational control input $\tau_{\omega }$; (**f**) r1 of case1; (**g**) r1 of case2; (**h**) r2 of case1 and case2.

Figure 6 shows the simulation results of case1 and case2.

It can be seen that case2 has better robustness than case1, so we choose the same control parameters as case2. The designed parameters of the inner loop controller are shown in Table 3.

Set $\omega_{d}\;=\;0.5\;$rad/s, $v_{d}\;=\;0.5\;$m/s as the control target for our inner loop controller. Then the initial state variables are supposed as ${\left[\delta (0)\;\omega (0)\;x_{v}(0)\;v(0)\;\theta (0)\;\dot{\theta }(0)\right]}^{T}=$ ${\left[0\;0\;0\;0\;0.1\;0\right]}^{T}$. In order to verify the robustness of the two controllers, the mass of the body is considered as a variable value in the simulation:

$$m=\left\{ \begin{array}{l} 2,t\leq 10\;s\\ 3,t>10\;s \end{array}\right.$$

**Figure 7:**
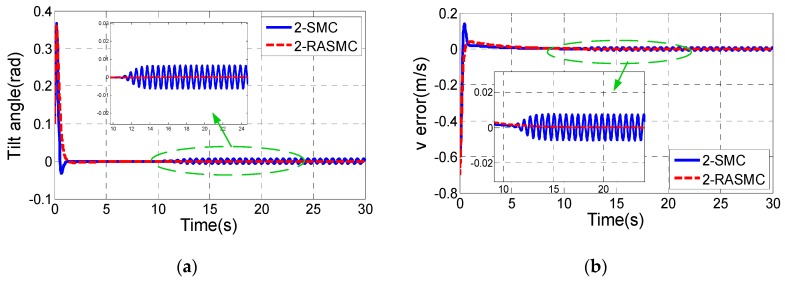

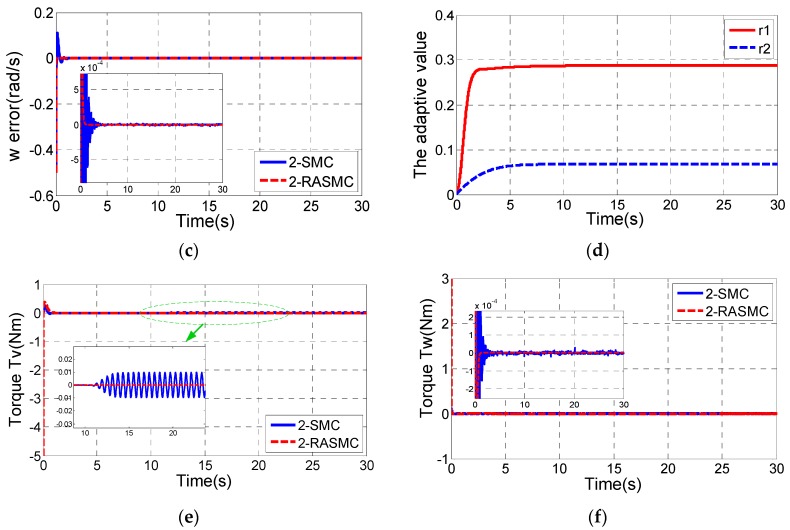
Comparison results of the two controllers with system uncertainty. (**a**) Tilt angle of the TWIP vehicle; (**b**) v tracking; (**c**) ω tracking ; (**d**) The adaptive value of 2-RASMC; (**e**) The longitudinal control input $\tau_{v}$; (**f**) The rotational control input $\tau_{\omega }$.

Figure 7 shows the simulation results.

Comparing Figure 7a,b,e with Figure 7c,f, it can be concluded that the robust performance of the control law of ω is much better than that of the control law of v in the 2-SMC controller. Considering Figure 7a,b,e, it can be easily seen that the state variables of the subsystems (8) and (9) in the 2-SMC controller oscillates near the equilibrium point after *t* > 10 s, while the control performance of the 2-RASMC controller is almost unaffected.

**Figure 8:**
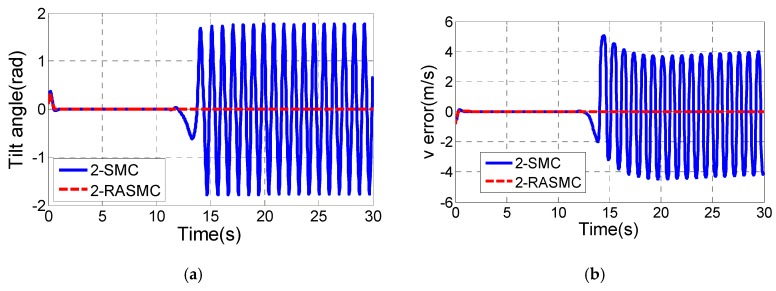

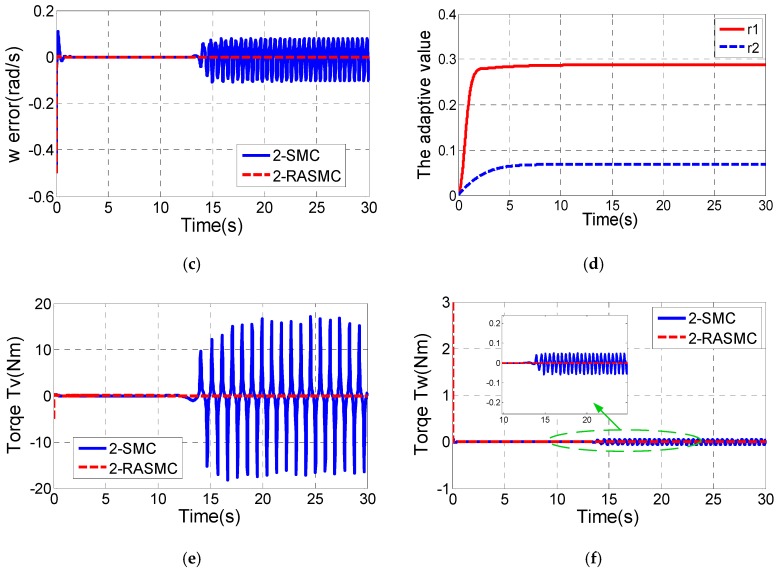
Comparison results of the two controllers with system uncertainty. (**a**) Tilt angle of the TWIP vehicle; (**b**) v tracking; (**c**) ω tracking ; (**d**) The adaptive value of 2-RASMC; (**e**) The longitudinal control input $\tau_{v}$; (**f**) The rotational control input $\tau_{\omega }$.

By increasing the value of m, the comparison of the robust performance of the two controllers will be more obvious. Set the value of m as $m=\left\{ \begin{array}{l} 2,t\leq 10\;s\\ 5,t>10\;s \end{array}\right.$, then the simulation result is shown as Figure 8.

From Figure 8, we can see the control performance of the 2-SMC controller becomes terrible after *t* > 10 s, while the 2-RASMC controller still maintains good control performance although the degree of system uncertainty is exacerbated. Combining Figure 7 and Figure 8, it can be observed that the 2-RASMC controller has better robustness.

### 4.2. Outer Loop Controller Design

**Figure 9:**
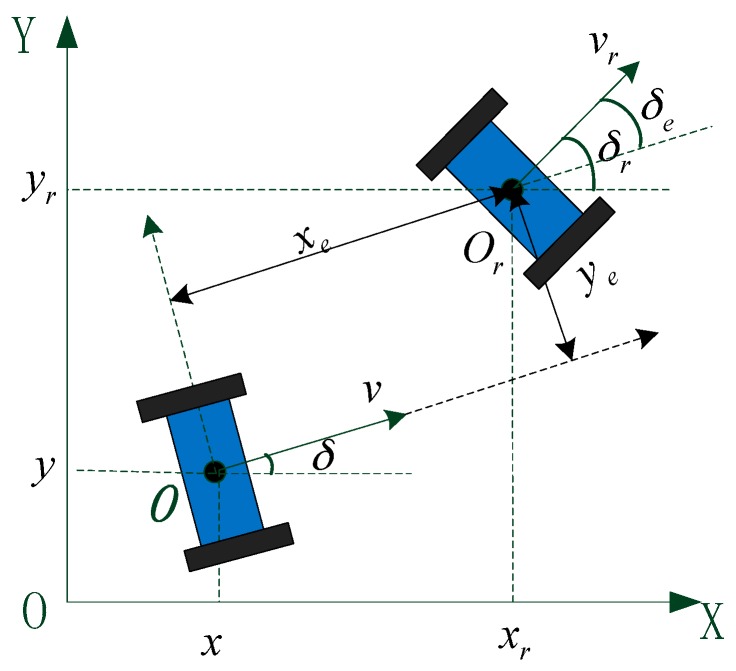
The posture errors of the two-wheeled vehicle.

The motion states of the TWIP vehicle on x-y plane are described by the center position of its wheel axis O (x, y) and the heading angle δ as shown in Figure 9. Defining $p={\left[x\;y\;\delta \right]}^{T}$, $q={\left[v\;\omega \right]}^{T}$, the kinematic equations of the vehicle can be obtained as:

(32) $$\dot{p}=\left[\begin{array}{l} \dot{x}\\ \dot{y}\\ \dot{\delta } \end{array}\right]=\left[\begin{array}{cc} \cos\delta & 0\\ \sin\delta & 0\\ 0 & 1 \end{array}\right]q$$

Defining $x_{r}{,\;y}_{r}{\;\mathrm{and}\;\delta }_{r}$ as the reference trajectory in the earth fixed coordinate system, then the reference longitudinal $v_{r}$ and rotational velocities $\omega_{r}$ can be calculated in prior respectively. The position and orientation errors are defined as:

(33) $$\left[\begin{array}{l} x_{e}\\ y_{e}\\ \delta_{e} \end{array}\right]=\left[\begin{array}{lll} \cos\delta & \sin\delta & 0\\ -\sin\delta & \cos\delta & 0\\ 0 & 0 & 1 \end{array}\right]\left[\begin{array}{l} x_{r}-x\\ y_{r}-y\\ \delta_{r}-\delta \end{array}\right]$$

Differentiating (33) with respect to time, the tracking error dynamics can be obtained as:

(34) $$\left[\begin{array}{l} {\dot{x}}_{e}\\ {\dot{y}}_{e}\\ {\dot{\delta }}_{e} \end{array}\right]=\left[\begin{array}{l} y_{e}\omega -v+v_{r}\cos\delta_{e}\\ -x_{e}\omega +v_{r}\sin\delta_{e}\\ \omega_{r}-\omega \end{array}\right]$$

Backstepping control method is often used to deal with underactuated stabilization. Backstepping has good tracking performance, fast response time without overshoot and is suitable for online control. Therefore, we design the outer-loop controller using the idea of backstepping.

Take $x_{e}$ as a virtual control variable, the new error variable is defined as:

(35) $${\bar{x}}_{e}=x_{e}-\lambda_{1}\tanh\left(\omega \right)y_{e}$$

When $\bar{x_{e}}\;\to \;0\;$ and $\delta_{e}\to 0$, according to Equation (35):

(36) $${\dot{y}}_{e}=-x_{e}\omega =-\lambda_{1}\omega \tanh\left(\omega \right)y_{e},\lambda_{1}>0$$

**Lemma** **1.** *For all* x ∈ R, *and the absolute value of x is bounded, there is* ϕ(x) = xtanh(x) ≥ 0, *if and only if x = 0 the equality holds.*

So, when t→ ∞, y_e_ exponentially converges to zero, thus x_e_→0.

*Adopting backstepping control method, the control law is deduced as follows:*

(37) $$\left\{ \begin{array}{l} w_{d}=w_{r}+2\lambda_{3}v_{r}y_{e}\cos\frac{\delta_{e}}{2}+\lambda_{4}\sin\frac{\delta_{e}}{2}\\ v_{d}=v_{r}\cos\delta_{e}+\lambda_{1}\tanh\left(\omega \right)\omega x_{e}-\lambda_{1}\tanh\left(\omega \right)\left(v_{r}\sin\delta_{e}+\lambda_{2}y_{e}\right)\\ \;+\lambda_{2}x_{e}-\lambda_{1}\left(1-\tanh^{2}\left(\omega \right)\right)\dot{\omega }y_{e} \end{array}\right.$$

*where, λ_1_, λ_2_, λ_3_ and λ_4_ are the designed positive constants.*

**Theorem** **5.** The control system (34) is stable, and the posture errors are asymptotically stable with the backstepping control law (37).

**Proof.**  First, define a Lyapunov candidate as:

(38) $$V=\frac{1}{2}{{\bar{x}}_{e}}^{2}+\frac{1}{2}{y_{e}}^{2}+\frac{2}{\lambda_{3}}\left(1-\cos\frac{\delta_{e}}{2}\right)$$

Differentiating Equation (38) with respect to time:

(39) $$\begin{array}{l} \dot{V}={\bar{x}}_{e}{\dot{\bar{x}}}_{e}+y_{e}{\dot{y}}_{e}+\frac{1}{\lambda_{3}}\sin\frac{\delta_{e}}{2}{\dot{\delta }}_{e}\\ \;={\bar{x}}_{e}[v_{r}\cos\delta_{e}-v-\lambda_{1}\left(1-\tanh^{2}\left(\omega \right)\right)\dot{\omega }y_{e}-\lambda_{1}\tanh\left(\omega \right)(v_{r}\sin\delta_{e}-x_{e}\omega )]\\ \;-\lambda_{1}\omega \tanh\left(\omega \right){y_{e}}^{2}+\frac{1}{\lambda_{3}}\sin\frac{\delta_{e}}{2}[\omega_{r}-\omega +2\lambda_{3}y_{e}v_{r}\cos\frac{\delta_{e}}{2}] \end{array}$$

Substituting the control law equations of ω and v (37) into Equation (39), then:

(40) $$\dot{V}=-\lambda_{2}{{\bar{x}}_{e}}^{2}-\lambda_{1}\omega \tanh\left(\omega \right){y_{e}}^{2}-\frac{\lambda_{4}}{\lambda_{3}}\sin^{2}\left(\frac{\delta_{e}}{2}\right)\leq 0$$

By LaSalle’s invariance principle [22,23] and the Lyapunov stability criterion [24], the stability of the outer-loop controller is proved. ☐

## 5. Simulation and Discussion

**Figure A1:**
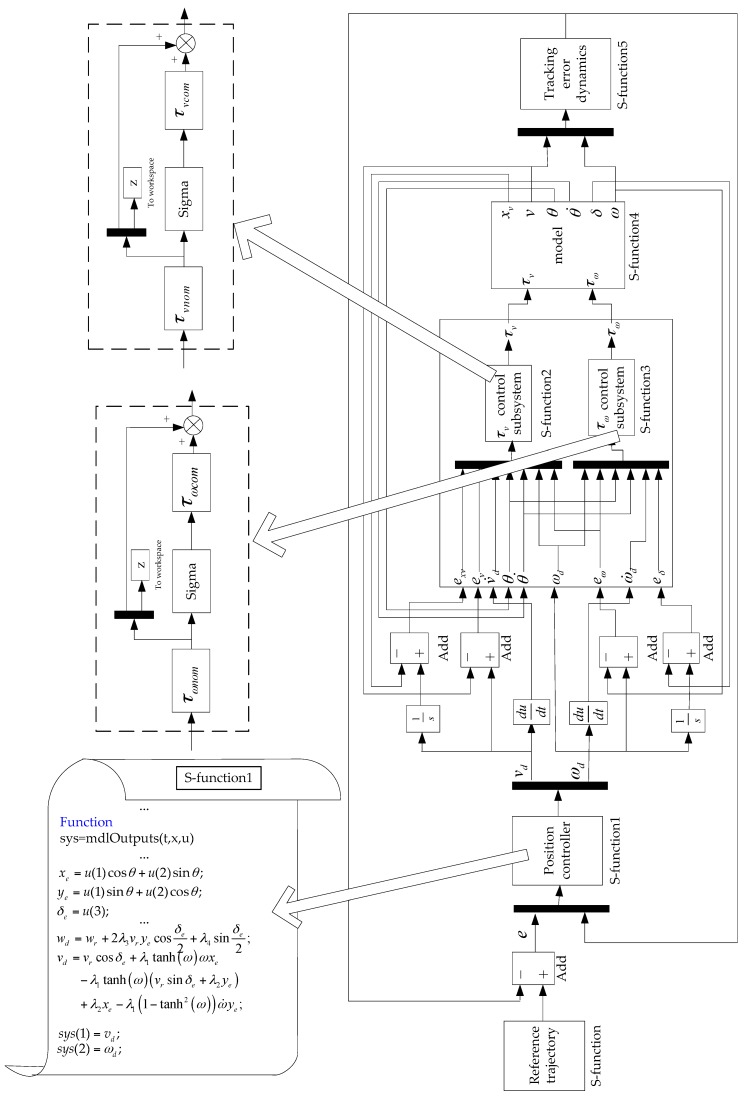
Simulation framework for 2S-B controller.

In this section, a lot of comparative simulations have been done to verify the validity of the proposed control methods. We have done a lot of simulations and found the appropriate control parameters of the designed outer loop controller as shown in Table 4. Table 5 exhibits several different algorithmic approaches combined by diverse control methods of inner and outer loop controllers. The simulation framework for 2S-B controller is shown in Figure A1 of the Appendix.

The control parameters of the PID controller used for comparison in this paper are settled by the Ziegler-Nichols method and shown in Table 6.

**Figure 10:**
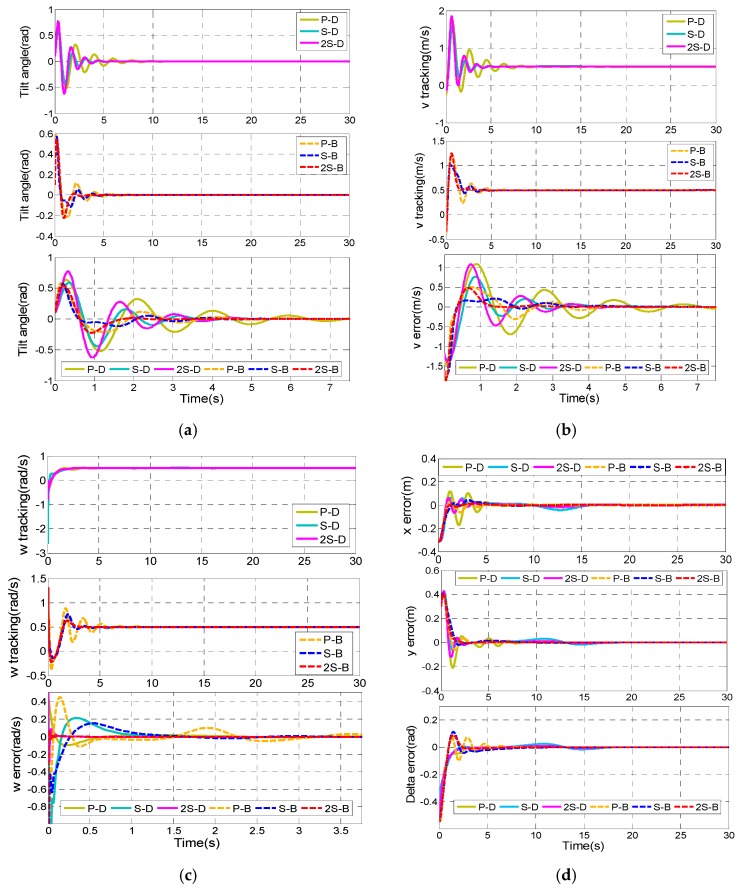

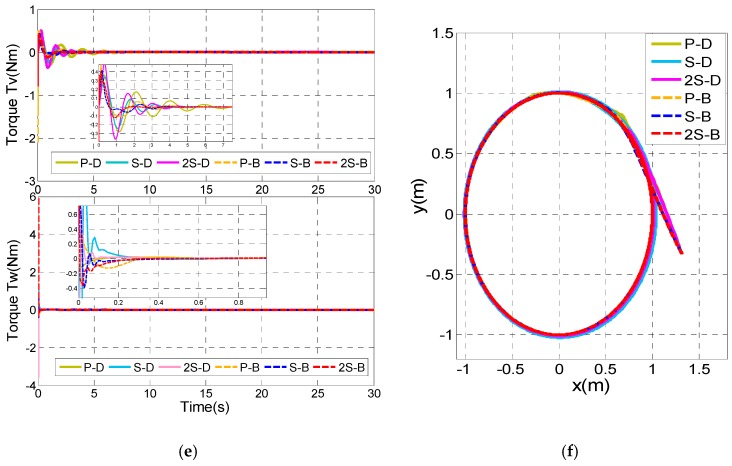
Comparison results of control combinations. (**a**) Tilt angle of the TWIP vehicle; (**b**) v tracking; (**c**) ω tracking ; (**d**) Tracking errors of x, y and δ; (**e**) Longitudinal control input $\tau_{v}$ and rotational control input $\tau_{w}$; (**f**) Tracking of the circle trajectory.

First, a reference circular trajectory with $\omega_{r}\;=\;0.5\;$ rad/s, $v_{r}\;=\;0.5\;$ m/s is given and the radius of the desired trajectory r is 1 m. Furthermore, we assume that the vehicle start point is ${\left[x(0)\;y(0)\;\delta (0)\right]}^{T}={\left[1.3\;-0.3\;2\pi /3\right]}^{T}$, and the initial state variables are supposed as ${\left[\delta (0)\;\omega (0)\;x_{v}(0)\;v(0)\;\theta (0)\;\dot{\theta }(0)\right]}^{T}={\left[0\;0\;0\;0\;0.1\;0\right]}^{T}$. Figure 10 shows the practical tracking for the circle mentioned above with six different control combinations.

From Figure 10a, it is clearly shown that the 2S-B controller has the shortest settling time of the tilt angle (about 1.55 s), while the worst settling time of the tilt angle is about 7.06 s in the P-D controller. The tracking error of x decreases to zero after t = 0.95 s by the 2S-B controller. When it comes to the P-D controller, the tracking error of x has the longest settling time of 4.44 s (Figure 10d). Moreover, the overshoots of θ and $e_{x}$ in the 2S-B controller are smallest among them. Figure 10d indicates that the DMLF method has better response about the tracking error of δ, but worse performances of $e_{x}$ and $e_{y}$ than the backstepping method. Considering these simulation results, the 2S-B controller is the most effective control combination strategy.

To further verify the effectiveness of the proposed controllers, a more sophisticated trifolium trajectory is used as a reference trajectory.

A trifolium trajectory can be designed as $x_{r}\left(t\right)\;=\;\rho \cos\left({3\omega }_{c}t\right)\cos\left(\omega_{c}t\right)$, $y_{r}\left(t\right)\;=\;\rho \cos\left({3\omega }_{c}t\right)\sin\left(\omega_{c}t\right)$. Here, the desired length of each leaf *ρ* is chosen as *ρ* = 5, $\omega_{c}$ = 0.05. Then, the reference longitudinal velocity $v_{r}\left(t\right)=\left|\sqrt{{{\dot{x}}_{r}}^{2}\left(t\right)+{{\dot{y}}_{r}}^{2}\left(t\right)}\right|$ and the reference rotational velocity $\omega_{r}(t)$ can be calculated in prior. Furthermore, we assume that the vehicle start point is ${\left[x(0)\;y(0)\;\delta (0)\right]}^{T}={\left[5.3\;-0.3\;0.6\pi \right]}^{T}$, and the initial state variables are the same as that of tracking a circle.

The system states are divergent for tracking a trifolium trajectory using the P-D controller and P-B controller. The reason why it diverges is the error of the longitudinal velocity $e_{v}$ converges more slowly than the acceleration of the reference longitudinal velocity $v_{r}$. Moreover, the simulation results are shown in the Appendix A.

**Figure 11:**
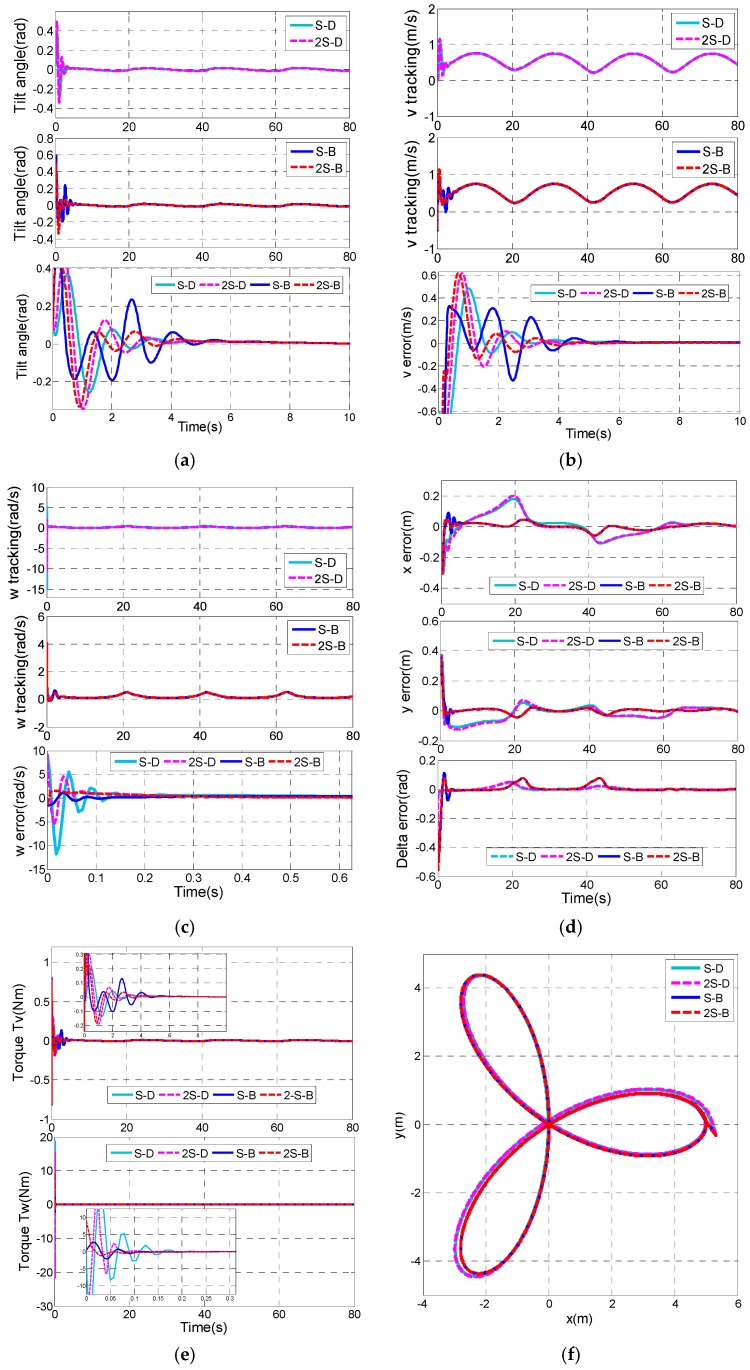
Comparison results of control combinations. (**a**) Tilt angle of the TWIP vehicle; (**b**) v tracking; (**c**) ω tracking ; (**d**) Tracking errors of x, y and δ; (**e**) Longitudinal control input $\tau_{v}$ and rotational control input $\tau_{w}$; (**f**) Tracking of the trifolium trajectory.

Figure 11 shows the comparison information of the four remaining control combinations, S-D controller, 2S-D controller, S-B controller and 2S-B controller.

It is clearly shown in Figure 11a that the S-D controller has the shortest settling time of the tilt angle (about 2.3 s), while the longest settling time of the tilt angle is about 4.37 s in the S-B controller. In Figure 11e, S-D and 2S-D have high rotational control torque $\tau_{w}$ up to around 20 Nm, while the rotational control torque $\tau_{w}$ in S-B controller is smaller than 2 Nm. The tracking errors of x and y are much smaller in the S-B controller and 2S-B controller than those of the S-D controller and 2S-D controller (Figure 11d). Moreover, the overshoots of $e_{x}$ and $e_{y}$ in the 2S-B controller are smallest among them.

Finally, a hexagon trajectory, which contains an abrupt variation of longitudinal velocity and rotational velocity, is more difficult to track than the trifolium one. The hexagon trajectory we adopt is described by piecewise linear functions. Furthermore, assume that the vehicle start point is ${\left[x(0)\;y(0)\;\delta (0)\right]}^{T}={\left[3.7\;-0.3\;3\pi /4\right]}^{T}$, and the initial state variables are set as ${\left[\delta (0)\;\omega (0)\;x_{v}(0)\;v(0)\;\theta (0)\;\dot{\theta }(0)\right]}^{T}={\left[0\;0\;0\;0\;0.1\;0\right]}^{T}$.

**Figure 12:**
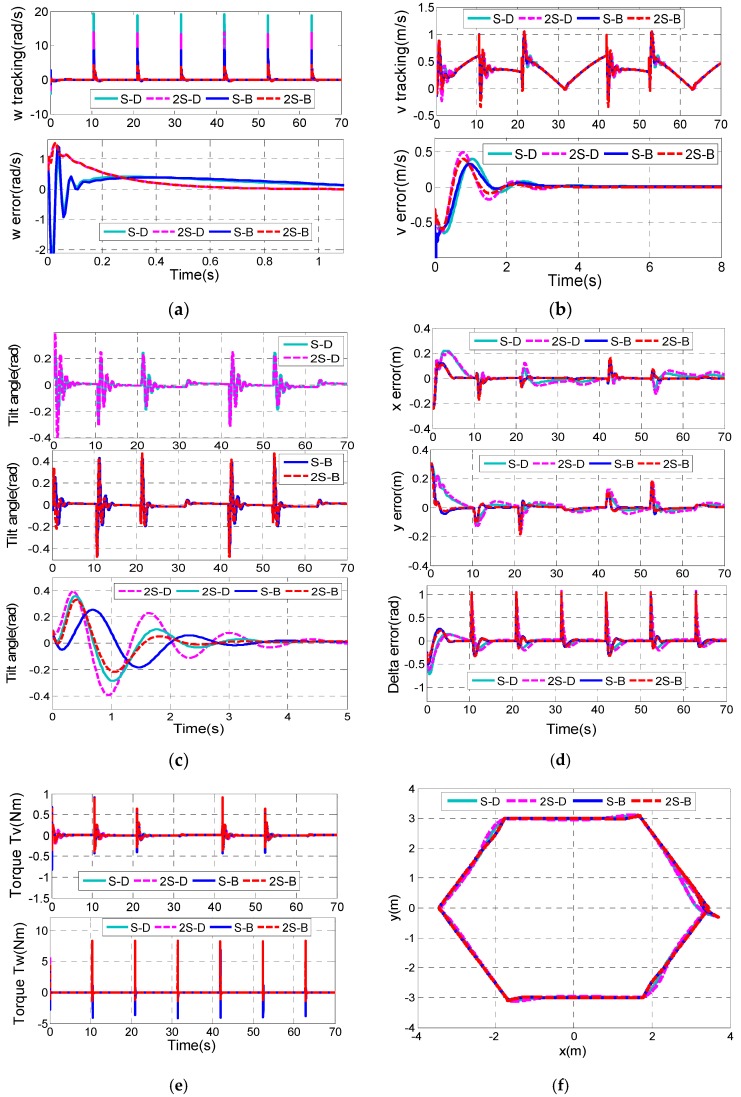
Comparison results of control combinations. (**a**) *v* tracking; (**b**) ω tracking ; (**c**) Tilt angle of the TWIP vehicle; (**d**) Tracking errors of x, y and δ; (**e**) Longitudinal control input $\tau_{v}$ and rotational control input $\tau_{w}$; (**f**) Tracking of the hexagon trajectory.

Similarly, the system states are divergent for tracking a hexagon using P-D controller and P-B controller. Moreover, the simulation results are shown in the Appendix A. Figure 12 shows the practical tracking performances for the hexagon mentioned above with four different control combinations.

Figure 12c shows that the 2S-B controller has the shortest settling time of the tilt angle (about 2.3 s), while the longest settling time of the tilt angle is about 3.3 s in the S-D controller. The tracking errors of x and y decrease most quickly to zero in the 2S-B controller. When it comes to the S-D controller and 2S-D controller, the settling time of $e_{x}$ and $e_{y}$ is up to about 7 s (Figure 12d). Moreover, the overshoots of $e_{x}$, $e_{y}$ and $e_{\delta }$ in the 2S-B controller are the smallest.

Although the hexagon trajectory seems to be tracked effectively, the torque performance is actually terrible due to the abrupt variation of the trajectory of longitudinal velocity and rotational velocity, and this situation is undesirable in practice.

Table 7 shows the different control effects of the six control combinations, such as the overshoot and the settling time of θ, $e_{x}$, $e_{y}$, $e_{\delta }$, and a coincidence factor indicator (CFI) which illustrates the trajectory tracking performance.

We define a coincidence factor indicator (CFI) to describe the tracking performance. The CFI is obtained as follows:

(41) $$\begin{array}{l} {\left(\sqrt{{e_{xi}}^{2}+{e_{yi}}^{2}}\right)}^{*}=\frac{\sqrt{{e_{xi}}^{2}+{e_{yi}}^{2}}-\min}{\max-\min}\\ CFI=\left(1-\frac{\sum_{i=1}^{N}{\left(\sqrt{{e_{xi}}^{2}+{e_{yi}}^{2}}\right)}^{*}}{N}\right)\times 100\% \end{array}$$

where, ()* means the normalization of expressions in brackets, min is the minimum of $\sqrt{{e_{xi}}^{2}+{e_{yi}}^{2}}$ from *i* = 1 to *i* = *N*, max is the maximum of $\sqrt{{e_{xi}}^{2}+{e_{yi}}^{2}}$ from *i* = 1 to *i* = *N*, N=T/0.05 is the number of samples, and T is the total time of simulation.

Different control effects of the six control combinations are clearly shown in Table 7, where we can see that the P-D and the P-B controller have poor adaptability to different trajectories. The convergence rates of $e_{x}$, $e_{y}$ and $e_{\delta }$ are much faster by using the backstepping method than using the DMLF method. It can be clearly seen that 2S-B method has the best CFI as well as shortest settling time of the tilt angle for all three trajectories, so it can be concluded that among the six control combinations, 2S-B controller has the best control performance for its fast convergence speed, small overshoot and best trajectory coincidence.

## 6. Experimental Results

**Figure 13:**
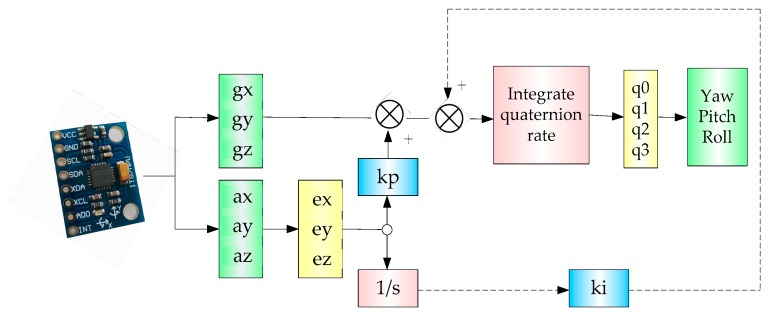
The frame of multi-sensor information fusion algorithm.

Since the sensor (MPU-6050) has an embedded 3-axis MEMS gyroscope and a 3-axis MEMS accelerometer, we need to utilize an information fusion algorithm to obtain relatively accurate information. Figure 13 shows the frame of the complementary filter for information fusion.

**Figure 14:**
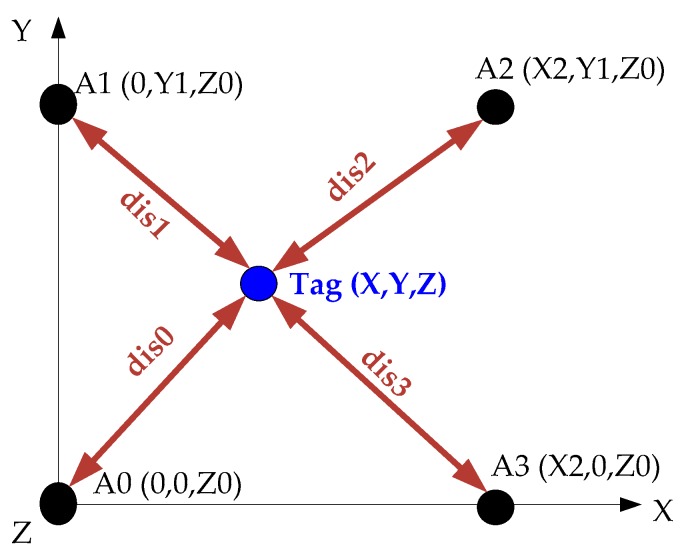
The placement of anchors in space.

The attitude data of the TWIP vehicle is measured via the onboard IMU, the velocity data is measured by two rotary encoders, and the location information of the TWIP vehicle is measured by the UWB positioning technology just as shown in Figure 2. The placement of anchors in space is shown in Figure 14.

The position of the Tag can be expressed as follows:

(42) $$\begin{array}{l} X=\frac{dis1^{2}-dis2^{2}+{X_{2}}^{2}}{2\times X_{2}}\\ Y=\frac{dis0^{2}-dis1^{2}+{Y_{1}}^{2}}{2\times Y_{1}} \end{array}$$

**Figure 15:**
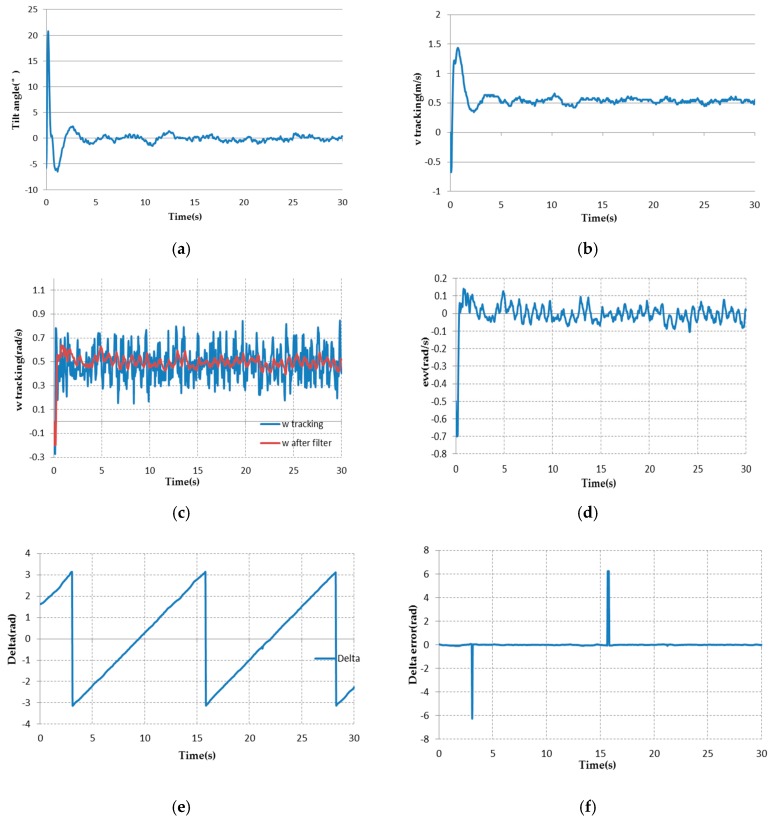

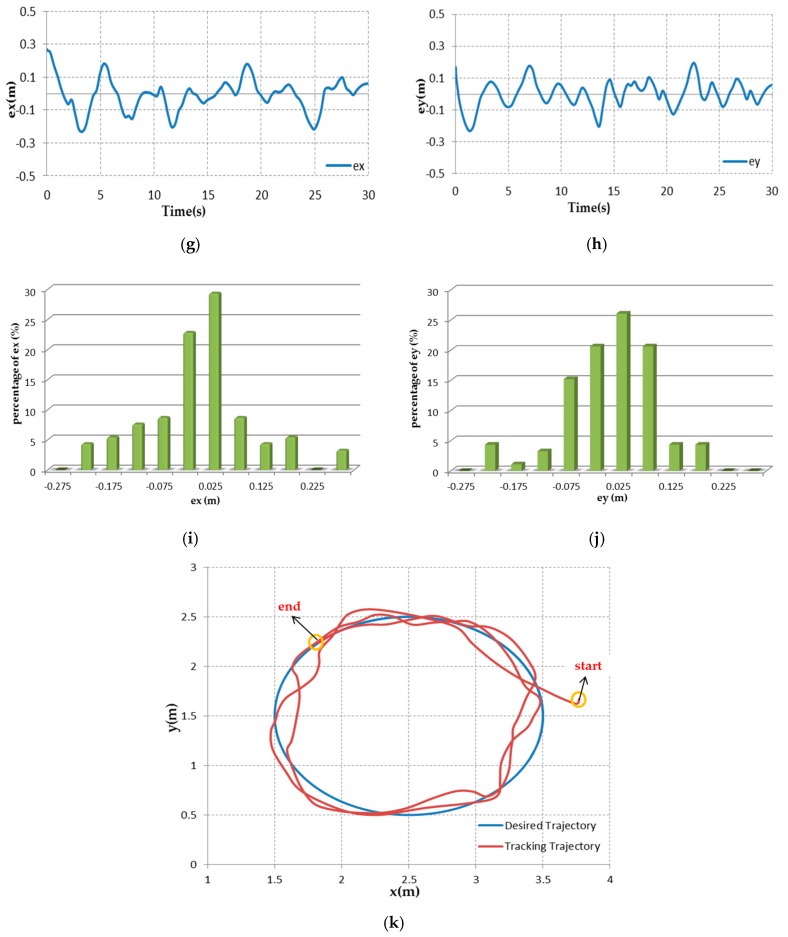
Experimental results of tracking for a circle. (**a**) Tilt angle of the TWIP vehicle; (**b**,**c**) v and ω tracking; (**d**) ω tracking error; (**e**) δ tracking; (**f**–**h**) Tracking errors of δ, x, y respectively; (**i**,**j**) Histograms of tracking errors of x, y respectively; (**k**) Practical tracking trajectory.

To verify the tracking performance of the proposed design, an indoor tracking test was implemented. The desired trajectory was set to be a circle with $\omega_{r}\;=\;0.5\;$ rad/s, $v_{r}\;=\;0.5\;$ m/s. The radius of the desired trajectory r was 1 m. Considering the existence of mismatch between the real-time system model and the mathematical model, the feedback gains obtained from simulations may not function well on the real-time platform, thus the control design parameters need to be adjusted through experiments on the prototype. In practice, we use the control design parameters as $c_{1}=5,\;c_{2}=0.4,\;c_{3}=4,\;\alpha =4,\;k_{1}=5,\;k_{2}=12,\;k_{3}=10,\;k_{4}=15,\;\gamma_{1}=1/3,\;\gamma_{2}=1/3,\;\lambda_{1}=0.8,\;\lambda_{2}=0.8,\;\lambda_{3}=20,\;\lambda_{4}=5.$ Figure 15 shows the indoor experimental results using the 2S-B controller for tracking the circle.

After the battery is fully charged, the TWIP vehicle can run continuously for about 30 min. The test of tracking a circle lasts about 30 s, and the TWIP vehicle finished the trajectory tracking for more than two rounds. Figure 15a shows the tilt angle of the TWIP vehicle. Figure 15g,h shows the position tracking errors. Because there is a cm-level measure error in the position measurement by UWB technology, the position tracking errors cannot converge to zero. The maximum position tracking error is about ±0.27 m for x-direction and about ±0.23 m for y-direction, but more than 80% of the all recorded horizontal points remain in a circle with a radius of 0.1 m. The RMS value of the position error is 0.1036 m in x-direction, and 0.0841 m in y-direction. The settling time of the tilt angle is about 3.5 s. Td the tilt angle is controlled within ±1.5°. This demonstrates the good tracking performance achieved in the indoor environment.

## 7. Conclusions

In this paper, an inner/outer loop control is proposed for the trajectory control of a TWIP vehicle. Several control methods have been utilized in the inner or outer loop controller and then different control combinations of inner and outer loop control methods have been proposed. A lot of simulations have been conducted to compare the control performance of different control combinations. Then, the simulation results illustrate that the 2S-B combination has the most effective control performance and highly adaptability to different trajectories. Finally experiments have been carried out to verify the effectivity of the 2S-B combination. In our future work, the Kalman filter and its variants will be considered to obtain the state estimation such that the effects of sensor noises can be attenuated. Further investigation will be conducted on path-planning arithmetic for environment exploration.

## Figures and Tables

**Table 1:** The parameters and variables of two-wheeled inverted pendulum vehicle system.

Parameter and Variable Name	Symbol	Value (Unit)
Acceleration due to gravity	g	9.8 (m/s^2^)
Mass of the wheel	M_W_	0.03 (kg)
Mass of the body	*m*	2 (kg)
Radius of the wheel	*r*	0.04 (m)
Length between the wheel axle and the center of gravity of the body	l	0.1 (m)
Rotational inertia of the wheel	J_W_	3.17 × 10^−5^ (kg·m^2^)
Rotational inertia of the body about the Y-axis	J_θ_	0.003 (kg·m^2^)
Rotational inertia of the body about the Z-axis	J_δ_	0.002 (kg·m^2^)
Distance between the two wheels along the axle center	D	0.17 (m)
Tilt angle of the vehicle body	θ	(rad)
The angle wheels turned	θ_r_, θ_l_	(rad)
Heading angle of the vehicle	δ	(rad)
Output torques of left and right wheel DC motors	τ_r_, τ_l_	(Nm)
Longitudinal displacement along the movement direction	X_V_	(m)
Longitudinal velocity of the vehicle	v	(m/s)
Rotational velocity of the vehicle	ω	(rad/s)

**Table 2:** The designed parameters of the 2-RASMC controller.

Control Parameter	$c_{1}$	$c_{2}$	$c_{3}$	α	$k_{1}$	$k_{2}$	$k_{3}$	$k_{4}$	$\gamma_{1}$	$\gamma_{2}$
**Case1**	5	0.4	4	4	5	12	10	15	3	3
**Case2**	5	0.4	4	4	5	12	10	15	1/8	1/3
**Case3**	5	0.5	3	3	3	5	10	15	3	3
**Case4**	5	0.5	3	3	3	5	10	15	1/8	1/3
**Case5**	0.5	0.5	3	3	3	5	3	5	3	3
**Case6**	0.5	0.5	3	3	3	5	3	5	1/8	1/3

**Table 3:** The designed parameters of the inner loop controller.

Control Parameter	$c_{1}$	$c_{2}$	$c_{3}$	α	$k_{1}$	$k_{2}$	$k_{3}$	$k_{4}$	$\gamma_{1}$	$\gamma_{2}$
**2-SMC**	5	0.4	4	4	5	12	10	15	--	--
**2-RASMC**	5	0.4	4	4	5	12	10	15	1/8	1/3

**Table 4:** The design parameters of the outer loop controller.

Design Parameters	$\lambda_{1}$	$\lambda_{2}$	$\lambda_{3}$	$\lambda_{4}$
**Value**	1	1	20	5

**Table 5:** Several algorithmic approaches of the inner/outer loop control system.

	Outer Loop Controller	Direct Method of Lyapunov Function (DMLF) [16]	Backstepping
Inner Loop Controller			
**PID**		P-D controller	P-B controller
**SMC [19]**		S-D controller	S-B controller
**2-RASMC**		2S-D controller	2S-B controller

**Table 6:** Control parameters of the PID controller.

Control Parameter	$k_{p\omega }$	$k_{i\omega }$	$k_{d\omega }$	$k_{pv}$	$k_{iv}$	$k_{dv}$	$k_{p\theta }$	$k_{i\theta }$	$k_{d\theta }$
**Value**	0.6	0.6	0.3	1.5	0	1.5	3	0	0.5

**Table 7:** The key performance indicators of three different control combinations.

Trajectory	Key Performance Indicators		DMLF			Backstepping		
			PID	SMC	2-SMC	PID	SMC	2-SMC
**Circle**	**θ**	**Overshoot σ (%)**	63.67	58.95	77.08	58.70	57.02	54.44
		**Settling time** $t_{s}$ **(s)**	7.06	3.40	3.81	4.01	3.22	1.55
	$e_{x}$	**Overshoot σ (%)**	11.75	6.03	5.75	4.52	4.08	0.96
		**Settling time** $t_{s}$ **(s)**	4.44	2.90	2.64	2.77	3.58	0.95
	$e_{y}$	**Overshoot σ (%)**	41.10	42.84	42.92	39.77	40.70	40.53
		**Settling time** $t_{s}$ **(s)**	5.23	1.45	1.87	2.57	1.44	1.34
	$e_{\delta }$	**Overshoot σ (%)**	0.19	2.38	0.95	7.23	11.21	8.96
		**Settling time** $t_{s}$ **(s)**	2.43	1.58	1.72	3.96	4.09	2.05
	**CFI (%)**		**97.22**	**95.80**	**97.54**	**97.83**	**96.64**	**98.39**
**Trifolium**	**θ**	**Overshoot σ (%)**	--	37.52	49.43	--	59.45	54.20
		**Settling time** $t_{s}$ **(s)**	--	2.30	2.63	--	4.37	3.02
	$e_{x}$	**Overshoot σ (%)**	--	17.96	19.90	--	9.01	4.80
		**Settling time** $t_{s}$ **(s)**	--	--	--	--	3.79	2.13
	$e_{y}$	**Overshoot σ (%)**	--	12.35	10.80	--	8.50	2.22
		**Settling time** $t_{s}$ **(s)**	--	24.59	25.87	--	2.38	0.82
	$e_{\delta }$	**Overshoot σ (%)**	--	0.00	0.00	--	11.32	7.65
		**Settling time** $t_{s}$ **(s)**	--	0.43	0.30	--	3.25	2.30
	**CFI (%)**		**--**	**88.51**	**86.13**	**--**	**94.88**	**95.65**
**Hexagon**	**θ**	**Overshoot σ (%)**	--	23.19	39.63	--	25.24	29.19
		**Settling time $t_{s}$ (s)**	--	3.27	3.30	--	2.63	2.33
	$e_{x}$	**Overshoot σ (%)**	--	21.93	20.90	--	11.97	11.48
		**Settling time$t_{s}$ (s)**	--	8.34	8.63	--	4.18	4.15
	$e_{y}$	**Overshoot σ (%)**	--	0.00	0.00	--	4.15	2.41
		**Settling time $t_{s}$ (s)**	--	6.98	7.47	--	4.29	1.95
	$e_{\delta }$	**Overshoot σ (%)**	--	15.31	13.39	--	26.85	22.57
		**Settling time** $t_{s}$ **(s)**	--	9.33	9.63	--	4.83	4.97
	**CFI (%)**		**--**	**89.08**	**87.31**	**--**	**95.41**	**96.12**

**Figure A2:**
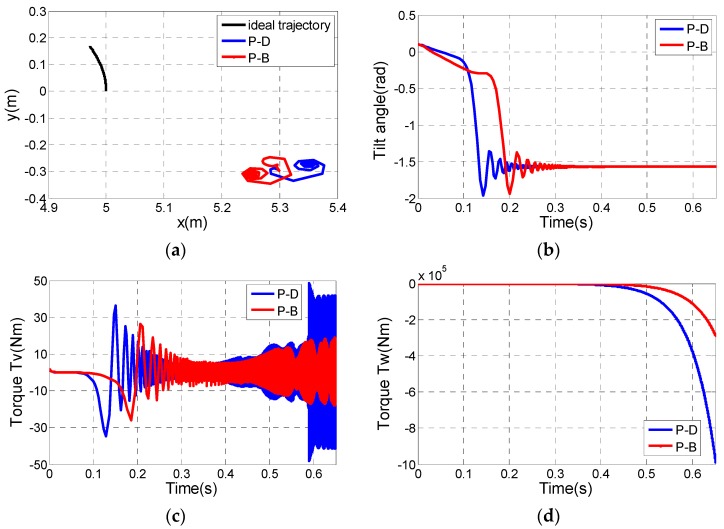
Control peformance of PID&Backstepping controller and PID&DMLF controller for tracking a trifolium, (**a**) Tracking trajectories; (**b**) Tilt angle of the TWIP vehicle; (**c**,**d**) Control torques $\tau_{v}$ and $\tau_{w}$.

**Figure A3:**
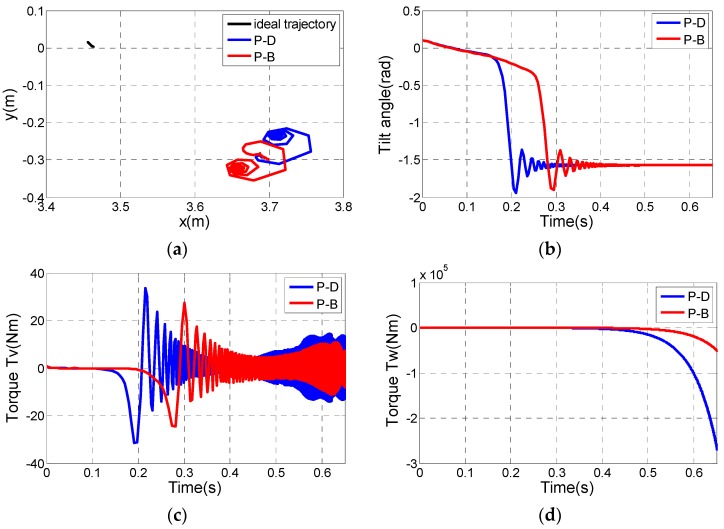
Control peformance of PID&Backstepping controller for tracking a hexagon, (**a**) Tracking trajectories; (**b**) Tilt angle of the TWIP vehicle; (**c**,**d**) Control torques $\tau_{v}$ and $\tau_{w}$.

## Appendix A

Figure A2a–d illustrate that the system states are divergent for tracking a trifolium trajectory using the P-D controller and P-B controller. Compared with the circle trajectory, the trifolium trajectory is more sophisticated and the tracking velocity $v_{d}$ changes rapidly at the beginning.

Figure A3a–d illustrate that the system states are divergent for tracking a hexagon using the P-D controller and P-B controller. Comparing with the circle trajectory, the hexagon trajectory is more sophisticated and the tracking velocity $v_{d}$ and $w_{d}$ changes rapidly at the beginning.
